# Microbiome signatures associated with clinical stages of gastric Cancer: whole metagenome shotgun sequencing study

**DOI:** 10.1186/s12866-024-03219-2

**Published:** 2024-04-24

**Authors:** Sohyun Jeong, Yi-Tyng Liao, Min-Hsuan Tsai, Yao-Kuang Wang, I-Chen Wu, Chung-Jung Liu, Ming-Shun Wu, Tze-Sian Chan, Ming-Yao Chen, Ping-Jen Hu, Wei-Yu Kao, Hsiang-Chin Liu, Ming-Ju Tsai, Cheng-Yuan Liu, Chun-Chao Chang, Deng-Chyang Wu, Yi-Hsiang Hsu

**Affiliations:** 1 Hinda and Arthur Marcus Institute for Aging Research, Hebrew SeniorLife, 1200 Centre Street, Boston, MA 02131 USA; 2 Department of Medicine, Beth Israel Deaconess Medical Center and Harvard Medical School, Boston, MA USA; 3 Development Center for Biotechnology, Taipei, Taiwan; 4 Broad Institute of MIT and Harvard, Cambridge, MA USA; 5 Division of Gastroenterology, Department of Internal Medicine, Kaohsiung Medical University Hospital, 100 TzYou 1st Rd, Kaohsiung City, 80756 Taiwan; 6 Department of Medicine, Faculty of Medicine, College of Medicine, Kaohsiung Medical University, Kaohsiung, Taiwan; 7 Regenerative Medicine and Cell Therapy Research Center, Kaohsiung Medical University, Kaohsiung, Taiwan; 8 Division of Gastroenterology and Hepatology, Department of Internal Medicine, Taipei Medical University Hospital, Taipei, 110 Taiwan; 9 Division of Gastroenterology and Hepatology, Department of Internal Medicine, School of Medicine, College of Medicine, Taipei Medical University, No.250, Wuxing St., Xinyi Dist, Taipei, 110 Taiwan; 10 TMU Research Center for Digestive Medicine, Taipei Medical University, No.252, Wuxing St., Xinyi Dist, Taipei, 110 Taiwan; 11 Division of Gastroenterology, Department of Internal Medicine, Wan Fang Hospital, Taipei Medical University, Taipei, Taiwan; 12 Division of Gastroenterology, Department of Internal Medicine, Shuang Ho Hospital, Taipei Medical University, Taipei, Taiwan; 13 Yourgene Health (Taiwan) Co., Ltd., Taipei, Taiwan

**Keywords:** Fusobacteria, Bacteroides_caccae, Bifidobacterium_longum, Streptococcus_anginosus, Lachnospiraceae_bacterium_5_1_63FAA, GLCMANNANAUT-PWY

## Abstract

**Background:**

Gastric cancer is one of the global health concerns. A series of studies on the stomach have confirmed the role of the microbiome in shaping gastrointestinal diseases. Delineation of microbiome signatures to distinguish chronic gastritis from gastric cancer will provide a non-invasive preventative and treatment strategy. In this study, we performed whole metagenome shotgun sequencing of fecal samples to enhance the detection of rare bacterial species and increase genome sequence coverage. Additionally, we employed multiple bioinformatics approaches to investigate the potential targets of the microbiome as an indicator of differentiating gastric cancer from chronic gastritis.

**Results:**

A total of 65 patients were enrolled, comprising 33 individuals with chronic gastritis and 32 with gastric cancer. Within each group, the chronic gastritis group was sub-grouped into intestinal metaplasia (*n* = 15) and non-intestinal metaplasia (*n* = 18); the gastric cancer group, early stage (stages 1 and 2, *n* = 13) and late stage (stages 3 and 4, *n* = 19) cancer. No significant differences in alpha and beta diversities were detected among the patient groups. However, in a two-group univariate comparison, higher *Fusobacteria* abundance was identified in phylum; *Fusobacteria* presented higher abundance in gastric cancer (LDA scored 4.27, *q = 0.041* in LEfSe). Age and sex-adjusted MaAsLin and Random Forest variable of importance (VIMP) analysis in species provided meaningful features; *Bacteria_caccae* was the most contributing species toward gastric cancer and late-stage cancer (beta:2.43, se:0.891, *p:0.008*, VIMP score:2.543). In contrast, *Bifidobacterium_longum* significantly contributed to chronic gastritis (beta:-1.8, se:0.699, *p:0.009*, VIMP score:1.988). Age, sex, and BMI-adjusted MasAsLin on metabolic pathway analysis showed that GLCMANNANAUT-PWY degradation was higher in gastric cancer and one of the contributing species was *Fusobacterium_varium*.

**Conclusion:**

Microbiomes belonging to the pathogenic phylum *Fusobacteria* and species *Bacteroides_caccae* and *Streptococcus_anginosus* can be significant targets for monitoring the progression of gastric cancer. Whereas *Bifidobacterium_longum* and *Lachnospiraceae_bacterium_5_1_63FAA* might be protection biomarkers against gastric cancer.

**Supplementary Information:**

The online version contains supplementary material available at 10.1186/s12866-024-03219-2.

## Background

Gastric cancer is one of the major health problems worldwide, ranking fifth in incidence and third in cancer-related mortality, as reported in the latest published global cancer statistics [1]. Long-term studies have confirmed that the development of gastritis with precancer lesions such as atrophic gastritis or intestinal metaplasia increases the risk of gastric cancer [2–4].

Gastritis diagnosis in clinical practice relies primarily on invasive endoscopy and histological examination [5], which cannot be performed frequently and easily. Hence, monitoring disease progression with non-invasive methods and detection of biomarkers are in high demand for prevention and treatment strategies for gastric diseases.

A series of studies have affirmed the role of microbiomes other than *Helicobacter pylori*, a well-known carcinogen [6], in gastric lesions [7–10]. In the gastrointestinal tract, trillions of microorganisms colonize the mucosal surface and lumen, constantly releasing immunomodulatory molecules that interact with and shape the immune system [11]. Analyses of alterations in gastric mucosa microbial changes at different stages of gastritis including superficial gastritis, atrophic gastritis, intestinal metaplasia, and gastric cancer found that shifts in gastric microbial composition are associated with progression toward a more advanced form of gastric disease [10, 12, 13].

Next-generation sequencing of fecal samples produced tens of millions of reads per sample, allowing for comprehensive analysis of both rare and abundant microbes with high genome sequencing coverage [14]. Furthermore, at this depth of sequencing, de novo prediction of genes is also possible [15].

Notably, the majority of microbiome studies on gastric diseases are limited to gastric mucosal samples utilizing 16S rRNA sequencing. In contrast, we performed whole metagenome sequencing to enhance the detection of bacterial species, examining both rare and abundant species, and employed multiple bioinformatics approaches to investigate the potential targets of the microbiome that could serve as indicators for distinguishing between chronic gastritis and gastric cancer.

## Materials and methods

### Study setting and sample

The study participants were recruited from the Kaohsiung Medical University Chung-Ho Memorial Hospital, as well as from multisite Taipei Medical University hospitals, which include Taipei Medical University Hospital, Wanfang Hospital, and Shuang-Ho Hospital. The participants were grouped into chronic gastritis (CG, *n* = 33) and gastric cancer (GCA, *n* = 32). Within these groups, further sub-groups were categorized: chronic gastritis without precancer lesions (non-intestinal metaplasia, NIM, *n* = 18), chronic gastritis with pre-cancer lesions (intestinal metaplasia, IM, *n* = 15), early-stage gastric cancer (Phase I and II, *n* = 13) and late-stage gastric cancer (Phase III and IV, *n* = 19). The cancer stage was clinically determined according to American Joint Committee on Cancer staging manual 8th Edition. The diagnosis of chronic gastritis with NIM, chronic gastritis with IM, and gastric cancer were confirmed through histological examination of endoscopic mucosal biopsies conducted by pathologists.

Exclusion criteria were participants with; any significant infectious disease requiring intensive antibiotic treatments within 6 months before fecal sample collection, a history of alcohol/substance dependence, any disease that needed immunosuppressant therapy, inflammatory bowel disease, indeterminate colitis, irritable bowel syndrome, colitis, persistent or chronic diarrhea of unknown etiology, and recurrent *Clostridium difficile* infection.

This study was approved by the Institutional Review Boards of Kaohsiung Medical University (IRB No. KMUHIRB-G(I)-20,200,024), Taipei Medical University (IRB No. N202108054), and Hebrew SeniorLife (IRB No. 2019–50) in Boston, MA, USA. All participants provided informed consent to participate in this study.

The study collected de-identified clinical and survey information from participants, only including data relevant to the research objectives. The procedures conducted in this study adhered to ethical standards established by the institutional and/or national research committees, following the principles of the 1964 Helsinki Declaration and its later amendments or equivalent ethical standards.

### Fecal sample collection and DNA extraction

The stool samples for the study were collected using OMNIgene-GUT tubes (OM-200, DNA Genotek). Each participant collected approximately 1 g of stool at home following the user instructions provided by the manufacturer. The collected samples were then returned to clinicians at Kaohsiung Medical University and Taipei Medical University hospitals. Since OMNIgene-GUT tubes do not need a cold chain, the collected samples were stored at room temperature for a period of up to 2 months (https://www.dnagenotek.com/ROW/products/collection-microbiome/omnigene-gut/OM-200.htm).

For DNA extraction, subsampled stool specimens of approximately 100 mg were processed using the QIAamp PowerFecal Pro DNA Kit from Qiagen (catalog number 51804) [16]. All lysis, separation of impurities and purification procedures adhered to the manufacturer’s protocol provided by the QIAamp PowerFecal DNA Kit. The QC criteria were applied to ensure the reliability of the extracted DNA. These criteria included a minimal DNA concentration of 30 ng/μl with no serious degradation observed by gel electrophoresis with a DNA fragment length over 1 kb and the total amount was higher than 300 ng.

### Whole metagenome shotgun sequencing (WMGS)

#### Next-generation sequencing library construction

Next-generation sequencing library preparations followed the protocol of the VAHTS Universal DNA Library Prep Kit for Illumina (ND607–01, Vazyme Biotech). For each sample, 200 ng genomic DNA was randomly fragmented to sizes less than 500 base pairs (bp) using a sonication method with an S220 Focused-ultrasonicator from Covaris. The fragments underwent End Prep Enzyme Mix for end repair, 5′ phosphorylation and dA-tailing in a single reaction, followed by T-A ligation to attach adaptors to both ends. The adaptor-ligated DNA was subjected to size selection using beads. and fragments of approximately 470 bp (with the approximate insert size of 350 bp) were recovered. Each recovered DNA was amplified by PCR using P5 and P7 primers, with both primers carrying sequences that can anneal with the flowcell to perform bridge PCR and P5/P7 primer carrying index allowing for multiplexing. The PCR products were purified using beads, validated and quantified by Qubit 3.0 Fluorometer (Cat No Q33216, Invitrogen).

The resulting library preparations were subjected to validation and quantification using a Qubit 3.0 Fluorometer (Cat No Q33216, Invitrogen). With the raw sequencing data, both the amount and the quality of the sequencing data were checked by the software Seqtk (v1.2-r94).

#### Covariate information

Clinical information used as covariates included age, sex, BMI, comorbidities (hypertension, dyslipidemia, and diabetes), and *Helicobacter pylori* infection/eradication history. Two different Food Frequent Questionnaires (FFQs) for recent diet information were collected along with fecal samples from all the subjects who participated in the study. The FFQ survey included checklists assessing recent and regular dietary habits about food types (fish, meat, vegetables, dairy products, etc.), food intake frequency, prebiotics, and probiotic use.

### Statistical analyses

#### Baseline characteristics of subjects

Baseline characteristics of subjects were expressed as mean ± standard deviation (SD), and frequency or proportion (percentage) and were compared using the unpaired Student t-test, and Fisher’s exact test or chi-square test, respectively. FFQs were tested by the Mann-Whitney U (Wilcoxon rank-sum) test between CG and GCA. To examine whether food consumption was associated with any differential microbiome features as well as any covariates, we performed Hierarchical All-Against-All Significance Testing (HAllA) [17].

#### Microbiome taxonomic and functional profiles

We used Whole Metagenome Shotgun (wmgx) workflow (https://github.com/biobakery/biobakery_workflows#whole-metagenome-shotgun-wmgx) in biobakery pipelines [18] to process the paired-end raw metagenome shotgun sequencing FASTQ files. The first step involved filtering low-quality or irrelevant reads from the metagenome shotgun sequencing data. This was done using the KneadData tool (version 0.70). Taxonomic profiles of shotgun metagenomes were generated using MetaPhIAn2 (version 2.7.8). MetaPhIAn2 utilizes a library of clade-specific markers to provide profiling of various taxonomic groups, including bacteria, archaea, eukaryotes, and viruses. Functional profiling was performed by HUMAnN2. HUMAnN2 constructed a sample-specific reference database based on the pangenome of a subset of the species detected by MetaPhlAn2 in the sample. This allowed for the determination of the abundance profiles of gene families (UniRef90s). The information on which species contributed to these genes was stratified by StrainPhlAn and could then be summarized into higher-level gene groupings. Protein-coding sequences in the constructed pangenomes were pre-annotated to their respective UniRef90 families [19]. UniRef90 represents a comprehensive and nonredundant protein sequence database.

#### Normalization and filtering process

Normalization plays a crucial role in differential abundance analysis, especially when dealing with metagenome sequencing data, as differences in sequencing depth can make read counts incomparable across samples. We used the total sum scaling (TSS) [20] that aimed to address the heteroscedasticity of the samples observed in the samples, thereby stabilizing the variance of the data [21] after removing archaeal and viral taxonomies in the samples. After normalizing the raw measures into relative abundances, we limited our analysis to only microbial features at each taxon level that were prevalent and abundant with mean relative abundance > 0.01% in at least 10% of the samples.

#### Microbiome community diversity

The Shannon index [22] was used to measure α-diversity. Alpha diversity assesses the diversity of species within a single sample or group of samples. To identify differences in alpha diversity between groups, an independent two-sample t-test was applied. β-diversity was computed using Bray-Curtis dissimilarity and summarized using weighted and unweighted principal coordinates analysis (PCoA) [23]. Statistical differences in beta diversity metrics between groups were tested by permutation multivariate analysis of variance (PERMANOVA).

#### Differentially abundant microbiome features

##### Multiple bioinformatics approaches

We employed a comprehensive approach to minimize the likelihood of false positive or false negative findings. For univariable association analysis, Wilcoxon-Sum Rank test, RNASeq (EdgeR) implementing empirical Bayes estimation, exact tests, generalized linear models and quasi-likelihood tests based on the negative binomial distributions [24], Linear discriminant analysis effect size (LEfSe) that can highlight features that are particularly relevant in distinguishing different classes or groups [25] were used. For multivariable analysis, backward stepwise multivariable generalized linear regression analyses were performed adjusting for age and sex utilizing MaAsLin (Multivariate Association with Linear Models) [26]. Additionally, Random Forest variable of importance (VIMP) was computed for feature selection using the randomforestSRC R package [27]. HAllA method was implemented to test correlation among all pairs of FFQ and species abundance. HALLA model tests for correlation among all pairs of variables in a high-dimensional dataset, and prioritizes statistically promising candidate variables. HAllA utilizes hierarchical false discovery correction to limit false discoveries and loss of statistical power attributed to multiple hypothesis testing.  All the comparisons were two-tailed and the False Discovery Rate (FDR) method was used for multiple testing corrections with adjusted *p* values (q values) in all approaches. All analyses were performed in the biobakery pipeline [18], Microbiomeanalyst platform [28] and R version 4.1.2.

## Results

### Baseline characteristics and FFQ

A total of 33 CG and 32 GCA fecal samples were collected. Within CG, there were NIM (*n* = 18) and IM (*n* = 15). Within GCA, there were early-stage GCA (*n* = 13) and late-stage GCA (*n* = 19). The mean age was younger in CG but was not significantly different. Sex, BMI, hypertension, dyslipidemia, diabetes, and *Helicobacter pylori* history (eradication history) were comparable between the CG and GCA groups (Supplementary Table 1).

Among the 44-food frequency questionnaire (FFQ) items, 5 items were significantly different between the two groups after multiple testing corrections (*q < .05*) (Supplementary Table 2). Notably, mushroom consumption displayed significant association with *Dorea_formicigenerans* and *Phascolarctobacterium_succinatutens* in HALLA clustering (*q = 0.007* and 0.010, respectively). Since both showed higher abundance in GCA in biomarker differential analysis in species, we removed these 2 species from the final results to prevent false positives from possible confounding effects of mushroom consumption.

### Microbiome community diversity

Microbiome community analysis for alpha (Shannon index) and beta diversity (PCoA) demonstrated no statistically significant difference in phylum, genus, or species between the 2-group comparison of CG and GCA. These results remained consistent when extended to the four-group comparison involving NIM, IM, early-stage GCA, and late-stage GCA (Supplementary Fig. 1 & 2). A whole taxa profile for 65 samples is available in Supplementary Table 3.

### Differential abundances

#### Phylum

In the Wilcoxon-Sum Rank test, *Actinobacteria* and *Fusobacteria* exhibited significantly different abundances between the two groups among 7 phyla. *Actinobacteria* demonstrated a higher abundance in individuals with CG*,* while *Fusobacteria* were more abundant in those with GCA (Table 1).

**Table 1:** Differential abundance by Wilcoxon Rank-Sum Test between chronic gastritis and gastric cancer in Phylum

Phylum	Median difference	Mean difference	Wilcoxon *p*-value
**Actinobacteria**	0.046	0.059	**0.025**
Bacteroidetes	−0.130	−0.099	0.059
Firmicutes	−0.045	0.024	0.759
**Fusobacteria**	0.000	−0.004	**0.006**
Proteobacteria	−0.003	0.020	0.655
Synergistetes	0.000	0.000	1.000
Verrucomicrobia	0.000	−0.001	0.724

Significant results are made in bold type.

**Fig. 1:**
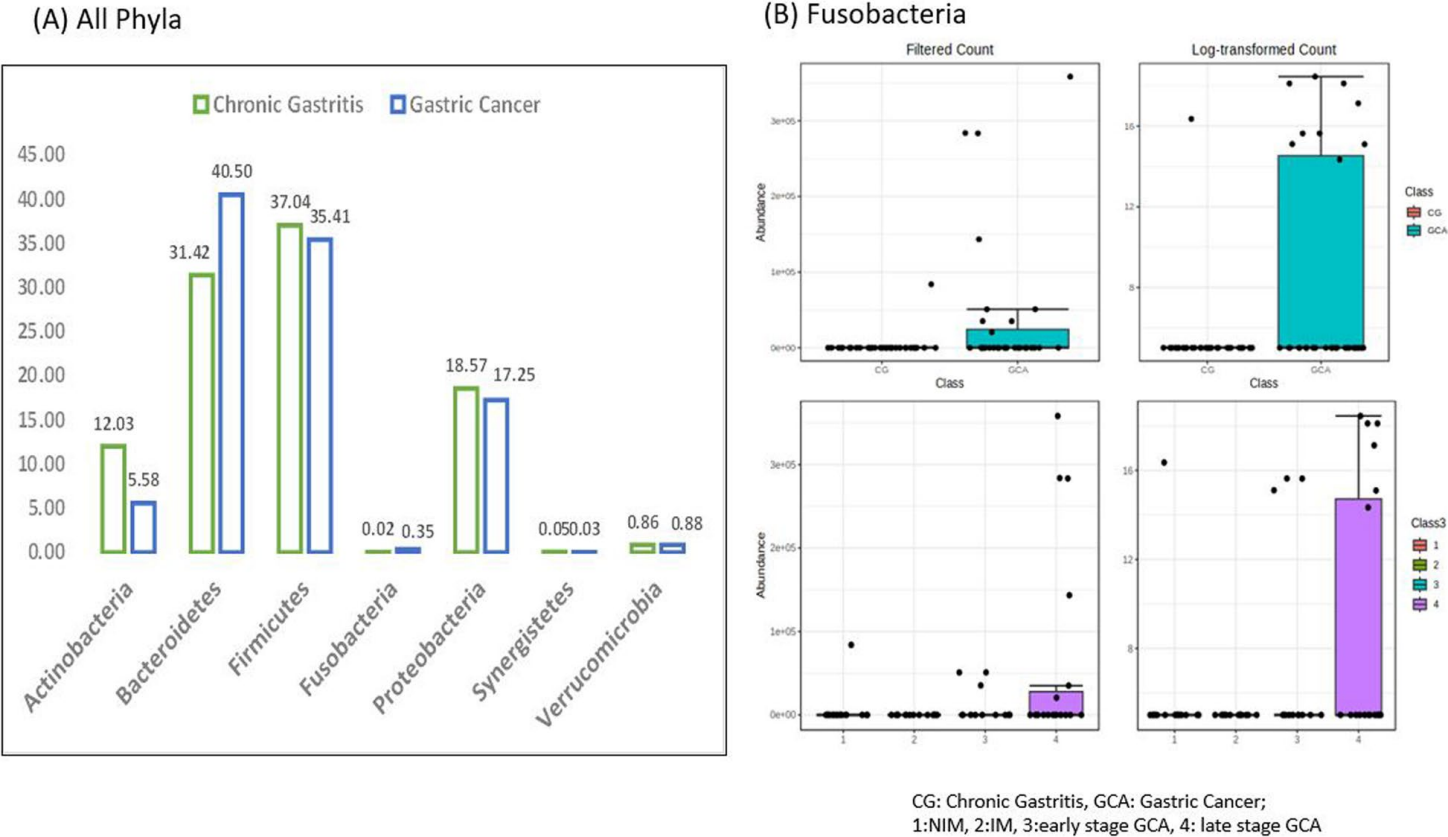
Differential abundance in phylum between chronic gastritis and gastric cancer. Footnote: (A) Y axis: relative abundance by percent value

In LEfSe analysis, for the 2-group comparison, only *Fusobacteria* was significantly higher in GCA after multiple testing corrections (*q =* 0.041, LDA score 4.27). In LEfSe analysis, among the 4-group comparison, *Fusobacteria* presented the highest abundance in the late-stage GCA but was not statistically significant (*q = 0.27*, LDA score 4.47) (Fig. 1 & Supplementary Table 4). Age and sex adjusted MaAslin model indicated that *Fusobacteria* was nominally significant (*p = 0.029*) with beta 0.685 (se 0.33) but multiple testing corrections turned to null (*q = 0.309*) (Supplementary Table 5).

#### Genus

**Fig. 2:**
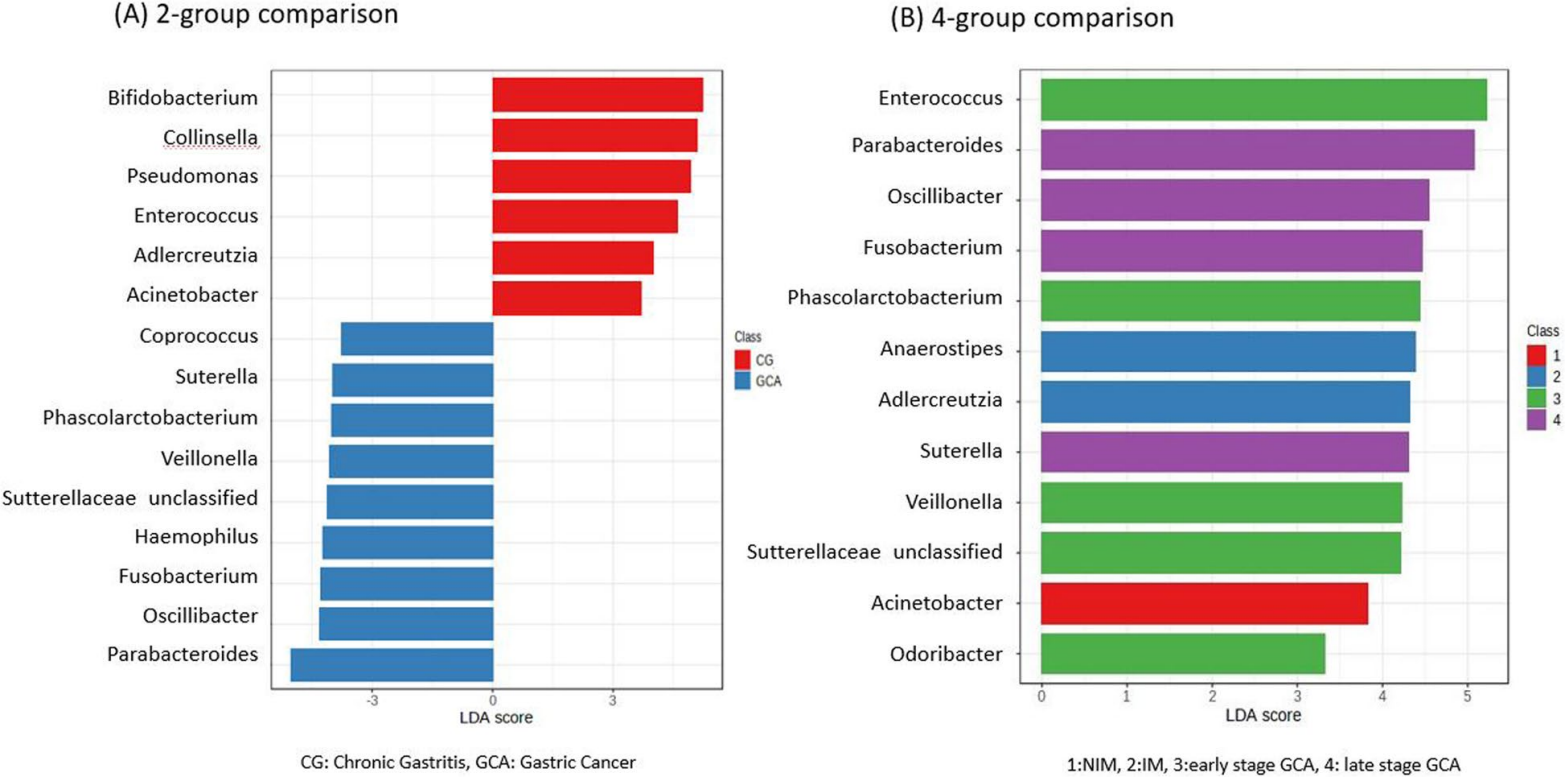
LEfSe analysis between chronic gastritis and gastric cancer in the genus

In the genus level analysis, 64 genera were used for final statistical analysis. Fifteen genera were significant in Wilcoxon-Sum Rank test. *Veillonella, Sutterellaceae_unclassified, Fusobacterium, Parabacteroides, Phascolarctobacterium, Sutterella,Oscillibacter, Haemophilus* and *Coprococcus*contributed to GCA whereas *Acinetobacter*, *Enterococcus, Adlercreutzia*, *Collinsella*, *Pseudomonas* and *Bifidobacterium* to CG (Supplementary Table 6). The same results were found in LEfSe analysis before multiple testing (Fig. 2 (A) & Supplementary Table 7). LEfSe analys in 4-group comparison showed *Acinetobacter* contributed to NIM, *Anaerotruncus* and *Adlercreutzia* to IM and *Fusobacterium*, *Oscillibacter* and *Parabacteroides* to late-stage GCA (Fig. 2 (B) & Supplementary Table 8).

In EdgeR analysis, *Eubacterium, Collinsella*, *Pseudomonas* and *Morganella* were significantly less but Lactobacillus is more abundant in GCA in the 2-group comparison. In 4-group comparison, *Megamonas* and *Pseudomonas* were more abundant in NIM whereas *Eubacterium* showed higher abundance in IM than the other groups (Table 2).

**Table 2:** Significant genera by EdgeR analysis

Name	Log2FC	LogCPM	Pvalues	FDR
2-group comparison				
Eubacterium	−1.471	15.17	9.021E-5	0.004
Lactobacillus	1.484	15.042	1.205E-4	0.003
Collinsella	−1.245	14.94	2.606E-4	0.005
Pseudomonas	−1.283	14.614	8.499E-4	0.012
Morganella	−1.176	14.654	0.002	0.029
4-group comparison				
Megamonas	−1.905	14.75	6.897E-4	0.040
Pseudomonas	−1.559	14.614	0.002	0.046
Eubacterium	1.449	15.17	0.002	0.046

In MaAsLin analysis (age and sex-adjusted), none of them surpassed multiple testing corrections but most of them remained nominally significant (*p < .05*) (Supplementary Table 9).

#### Species

**Fig. 3:**
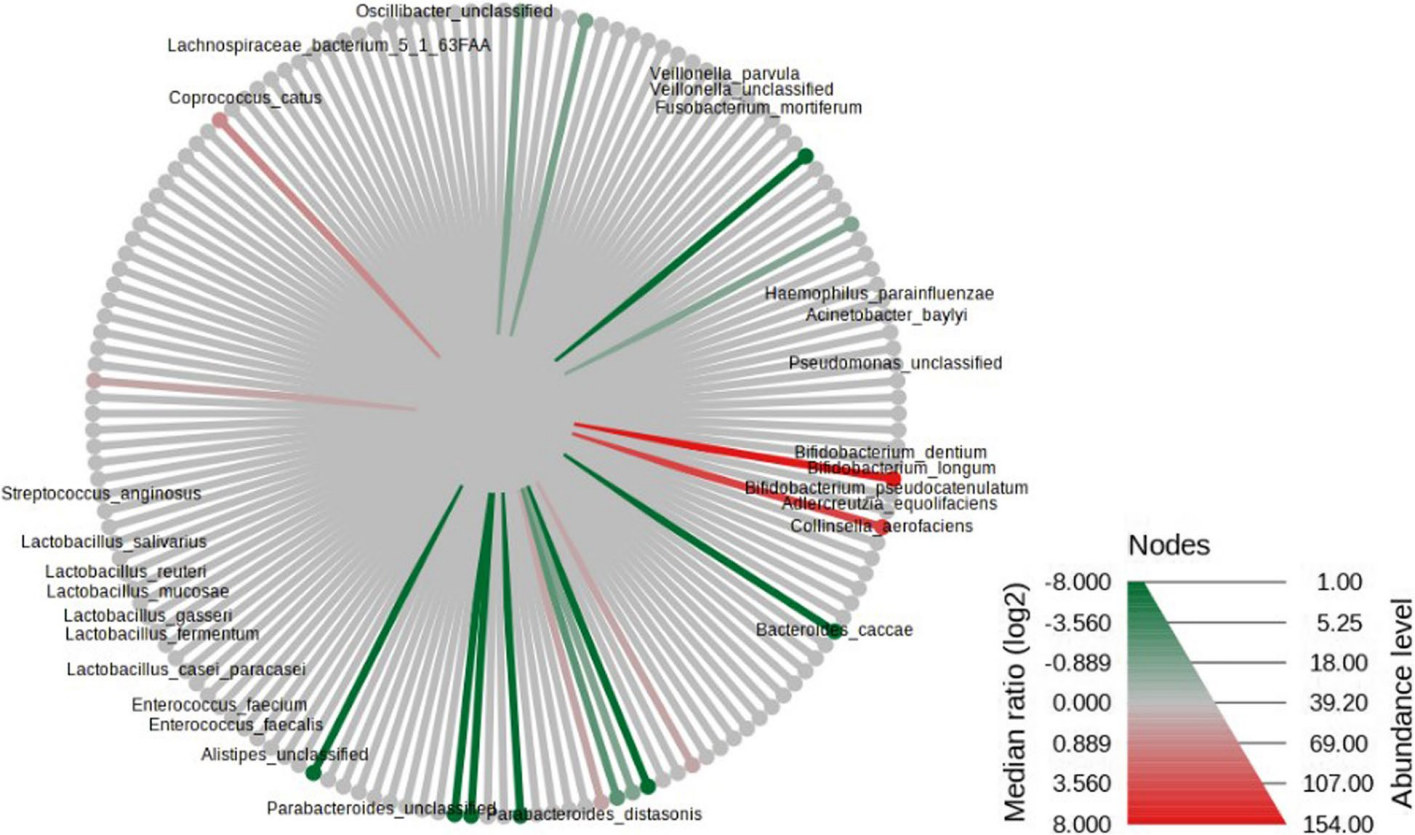
Heat Tree by Wilcox-Rank Sum test. Footnote: Red color means higher abundance in chronic gastritis (CG). Green color means higher abundance in gastric cancer (GCA)

After filtering low-abundance species (refer to normalization and filtering process in method section), 156 species remained for further analysis. In the Wilcoxon Rank Sum test, 29 species had significantly different abundances between CG and GCA. *Fusobacterium_mortiferum* is among one of them (*p = 0.02*) (Fig. 3). *Dorea_formicigenerans* and *Phascolarctobacterium_succinatutens* that showed significant association with mushroom consumption in HALLA analysis also presented significance, we removed these 2 species in a later analysis (Supplementary Table 10). Using the Random Forest algorithm, classification of CG and GCA showed modest out of bag (OOB) error rates of 21.9, and 27.3%, respectively. In 4 group classifications, Random Forest presented high OOB error, NIM (66.7%), IM (60%), early-stage GCA (100%), and late-stage GCA (42.1%) (Supplementary Fig. 3).

**Fig. 4:**
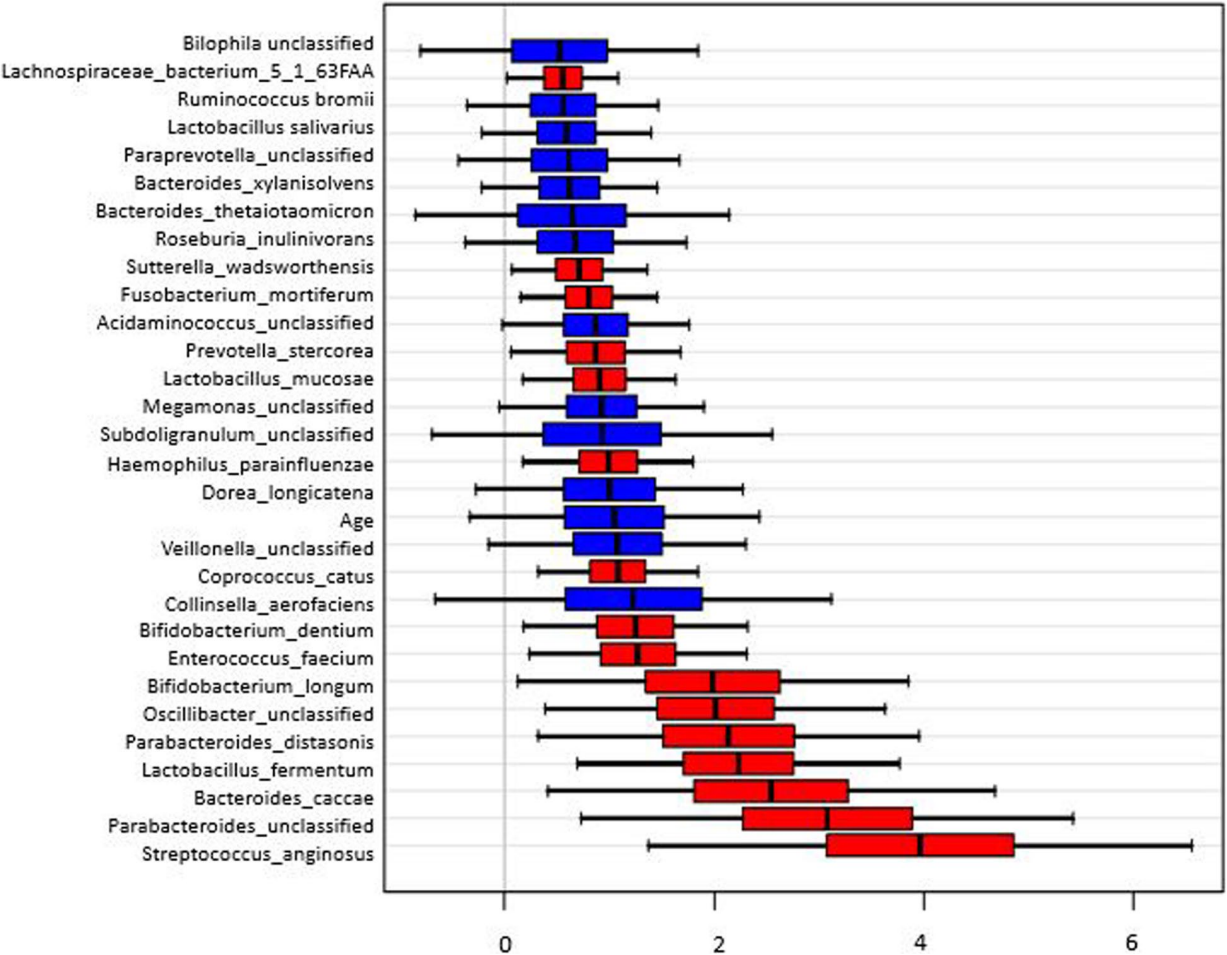
Top 30 features by Variable importance by Random Forest. Footnote: Red-colored bars are significant species within 95% CI of VIMP scores

**Fig. 5:**
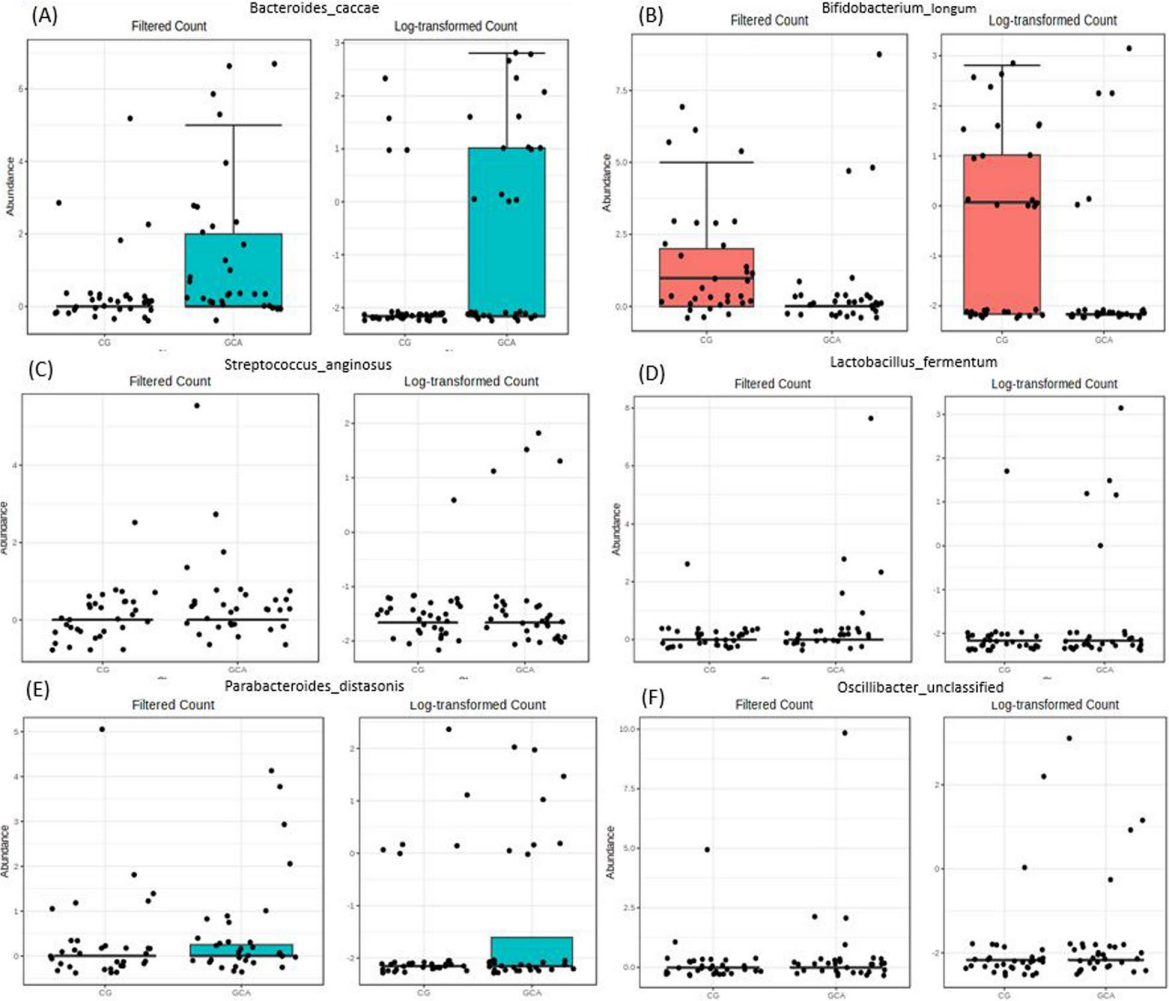
Important species between chronic gastritis and gastric cancer by MaAsLin and VIMP (VIMP score > 2) **A** Bacteroides_caccae **B **Bifidobacterium_longum **C** Streptococcus_aginosus **D** Lactobacillus_fermentum **E** Parabacteroides_distasonis **F** Oscillibacter_unclassified

In the age and sex adjusted MaAslin analysis in species, *Bacteria_caccae* was the most significant species among a total of 16 nominally significant species (*p < .05*) but none of them surpassed the multiple testing (Supplementary Table 11). Additional validation through the Random Forest VIMP (Fig. 4 & Supplement Table 12) analysis, 11 species were finally selected as important features having significant VIMP scores above 10 as well as significant findings from MaAsLin results (Fig. 5 & Table 3). *Bifidobacterium_longum, Enterococcus_faecium* and *Lachnospiraceae_bacterium_5_1_63FAA* showed higher abundance in CG compared to GCA whereas *Bacteroides_caccae, Bifidobacterium_dentium, Streptococcus_anginosus, Coprococcus_catus, Lactobacillus_fermentum, Parabacteroides_distasonis, Oscillibacter_unclassified* and *Lactobacillus_mucosae* presented higher abundance in GCA (Fig. 5 & Table 3).

**Table 3:** Important species differentiating CG and CGA

	MaAsLin			Random Forest VIMP	
Species	Beta	SE	*Pvalue*	VIMP score	95% CI
Bacteroides_caccae	2.43	0.891	0.008	2.543	0.905–4.182
Bifidobacterium_longum	−1.8	0.669	0.009	1.988	0.315–3.661
Bifidobacterium_dentium	0.688	0.265	0.012	1.249	0.522–1.976
Streptococcus_anginosus	1.18	0.473	0.015	3.966	3.802–4.850
Coprococcus_catus	0.36	0.147	0.017	1.081	0.746–1.416
Lactobacillus_fermentum	1.36	0.569	0.020	2.230	1.578–2.883
Parabacteroides_distasonis	1.36	0.577	0.022	2.135	0.769–3.502
Enterococcus_faecium	−0.576	0.253	0.026	1.272	0.792–1.753
Lachnospiraceae_bacterium _5_1_63FAA	−0.786	0.35	0.028	0.556	0.300–0.812
Oscillibacter_unclassified	1.33	0.621	0.036	2.012	0.954–3.071
Lactobacillus_mucosae	0.406	0.194	0.040	0.906	0.425–1.387

**Fig. 6:**
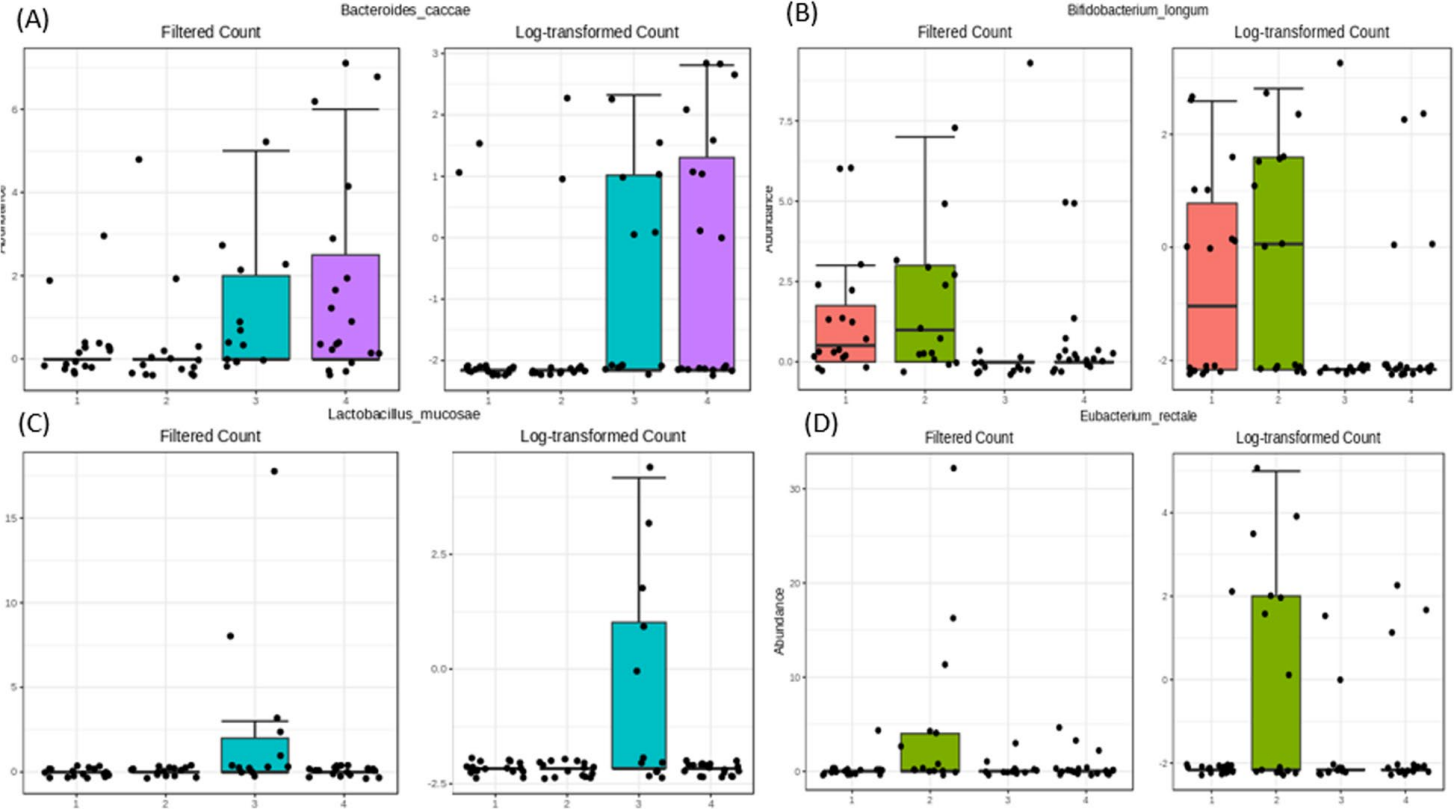
Distribution of microbiome abundance across 4 groups in important 4 species **A **Bacteroides_caccae **B **Bifidobacterium_longum **C** Lactobacillus_mucosae **D **Eubacterium_rectale

EdgeR analysis among the 4-group comparison, *Eubactrium_rectale* was significantly higher in IM (logFC 2.120, SE: 14.127, *q = 0.002*). We also explored 4 group comparisons in important features found in Table 3. Although they were not statistically significant in EdgeR results, *Bacteroides_caccae* presented a higher abundance in late-stage GCA, whereas *Bifidobacterium_longum* showed a higher abundance in IM, and *Lactobacillus_mucosae* showed a higher abundance in early-stage GCA (Fig. 6).

#### Metabolic pathway analysis

Age, sex and BMI adjusted MasAsLin analysis indicated that the superpathway of L-lysine, L-threonine and L-methionine biosynthesis I and II, superpathway of pyrimidine ribonucleosides salvage and superpathway of N-acetylglucosamine, N-acetylmannosamine and N-acetylneuraminate degradation were more enriched in GCA. In particular, GLCMANNANAUT-PWY; superpathway of N-acetylglucosamine, N-acetylmannosamine and N-acetylneuraminate degradation was associated with *Fusobacterium_varium* (Supplementary Table 13).

## Discussion

The overall composition and community diversities of the microbiome were similar between the CG and GCA groups irrespective of the specific subgroups within these categories. These results align with previous findings reported in other studies [29, 30].

In particular, we found that an enrichment of microbiota belonging to the phylum *Fusobacteria* was significantly associated with GCA, which has been confirmed in multiple studies [29, 31–33]. The genus *Fusobacterium* was fre*q*uently abundant in patients with gastric cancer, and a receiver operating characteristic curve analysis revealed that species *Fusobacterium_nucleatum* exhibited a diagnostic ability for gastric cancer [29]. The distribution of genus *Fusobacterium* in tumor tissues was demonstrated [31]. *Fusobacterium_nucleatum*, which originates from the oral cavity, can potentiate the carcinogenesis of colorectal cancer involving the activation of Wnt target genes which increase the secretion of proinflammatory cytokines and evade anticancer immune response [32, 33]. Hsieh et al have shown that *Fusobacterium_nucleatum* colonization leads to a worse prognosis in GCA patients with *H. pylori* positivity [29]. However, we didn’t detect *Fusobacterium_nucleatum* from fecal samples in our study, instead, we detected *Fusobacterium_mortiferum* and *Fusobacterium_varium* by WMGS. This might be due to the sample difference, most of the previous study findings for *Fusobacterium_nucleatum* were from samples collected from stomach tissues. *Fusobacterium_mortiferum* was significantly enriched in the GCA group in univariate analysis and *Fusobacterium_varium* was significantly associated with the microbial metabolic pathway of GLCMANNANAUT-PWY which was highly enriched in the GCA group in our study. Despite limited reports about *Fusobacterium_varium*, in a study among the patients with *Fusobacterium* infections in Korea, patients with *Fusobacterium_varium* infections were older and had a higher proportion of nosocomial infections than the other groups. The *Fusobacterium nucleatum* and *Fusobacterium_varium* groups showed higher in-hospital mortality than the other patients with *Fusobacterium* species [34]. *Fusobacterium_varium* as well as species belonging to *Fusobacteria* might be potential targets to study in the future in microbiome research.

As well-known pathogenic species, *Clostridium perfringens*, *Clostridium perfringens 13*, *Clostridium perfringens A99* and *Escherichia coli K-12 substr.* also share GLCMANNANAUT-PWY. These species can generate carbon and nitrogen sources through these N-acetylglucosamine, −mannosamine and -neuraminic acid degradation pathways [35] which might provide sources of N-nitroso compound (NOC) in affected patients.

It is known that patients with GCA have higher NOC levels than healthy subjects [36]. Genera *Veillonella* and *Lactobacillus* which were found significantly high in the GCA group in our genus analysis, contributed to gastric carcinogenesis by stimulating the production of NOCs [37, 38]. *Veillonella* was significantly lower in gastritis subjects than in gastric adenoma or advanced gastric cancer subjects in the previous study [39].

 At the species level, we also found a few protective species that were more abundant in CG than GCA*. Bifidobacterium longum* were higher in CG and according to the previous study, they were more abundant in the cancer patients who responded well to chemotherapy than non-responders [40]. *Bifidobacterium_longum* strains regulate oxidative stress by regulating the production and accumulation of ROS (reactive oxygen species), thereby reducing the symptoms of Inflammatory Bowel Disease [41]. *Lachnospiraceae_bacterium_5_1_63FAA* belongs to *Lachnospiraceae* which has been linked to protection from colon cancer in humans, mainly due to the association with the production of butyric acid, a substance that is important for both microbial and host epithelial cell growth [42].

 Whereas a few pathogenic species were also detected. Among them,* Bacteroides_caccae, a pathogenic species previously found in cultures from infections in the appendix and the peritoneal abdomen [*43] was higher in GCA.

*Bacteroides*_*caccae* degraded the mucus [44], which would lead to a condition of a “leaky gut” and therefore increased the permeability of the intestinal barrier. Clinical and experimental data suggested the importance of intestinal hyperpermeability in the inflammatory changes of various diseases including GI cancers [45]. *Streptococcus_anginosus* showed higher habitation in gastric tumors in the previous study [46]. *Oscillibacter* has been positively correlated with gut permeability [47].

However, some of the results are controversial from the previous findings. *Bifidobacterium_ dentium*, appeared to protect mucin glycans which is vital in the gut barrier [48]. *Lactobacillus_fermentum* UCO-979C is a good probiotic for the protection against *H. pylori* infections [49]. *Enterococcus_faecium* is the main causative agent of infection in humans and frequently demonstrates resistance to vancomycin, ampicillin, and other antimicrobials [50]. *Coprococcus* was less abundant in colon cancer compared to healthy individuals, although there was no evidence for its protective role against colon cancer [51]. *Parabacteroides_distasonis* attenuates toll-like receptor 4 signaling and Akt activation and blocks colon tumor formation in high-fat diet-fed azoxymethane-treated mice [52]. *Lactobacillus mucosae* has been reported as cardio-protective [53].

Based on these findings, we assume CG can also present a degree of gastric disease related microbial perturbations which can result in higher abundance of pathogenic microbiota found in CG as well.

In this study, we detected species-level microbiome markers and associated metabolic pathways by using WMGS, which enabled us to find novel species belonging to *Fusobacterium* known to be associated with poor prognosis of gastric diseases or gastric cancer. We also applied multiple bioinformatics approaches encompassing various patient characteristics and food consumption history which would affect the microbiome composition and delineate potential microbiome signatures that could be utilized as diagnostic or treatment biomarkers.

We also have limitations to address. First, due to the small sample size, the generalization of these results would be limited. However, multiple previous studies generated similar results, especially in the Asian population, which might validate this study’s results. Second, most study findings did not surpass multiple testing correction but we incorporated Random Forest VIMP scores to add further evidence to species found in MaAsLin multivariable models. We assume this study added clinically meaningful new findings to the previous microbiome studies in the gastric disease area. Third, medication use might have affected the gut microbial composition, however, due to the lack of information on medication use, we could not adjust this factor in the multivariable model. Fourth, *Helicobacter_pylori* is a well-established risk factor for gastric cancer, and it is worthwhile to investigate the association between *Helicobacter_pylori* and other microbiome features. However, we used fecal samples in this study, and *Helicobacter_pylori* which usually inhabits the upper gastric region was not detected, which limited additional exploration on *Helicobacter pylori*. Finally, although we sub-grouped the patients in order to dissect the different microbiome features corresponding to different stages of gastric diseases, the cross-sectional approach itself has limitations portraying only a snapshot of the time we collected the fecal samples. Therefore, designing longitudinal time-varying fecal sampling approaches within individuals is warranted.

## Conclusion

CG and GCA share similar microbial community characteristics. However, several distinctive microbiome pathogenic features including *Fusobacteria*, *Bacteroides_caccae,* and *Streptococcus_anginosus* might be represented as signature indicators for the progression of CGA. In addition, *Bifidobacterium_longum, and Lachnospiraceae_bacterium_5_1_63FAA* might be protective biomarkers against advanced gastric diseases.

### Supplementary Information

**Supplementary Material 1.**

**Supplementary Material 2.**

**Supplementary Material 3.**

**Supplementary Material 4.**

## Data Availability

Metagenomic raw sequencing data have been deposited in SRA database with BioProject ID PRJNA1065874. Raw data can be accessed here: https://www.ncbi.nlm.nih.gov/bioproject/PRJNA1065874

## Abbreviations

CG: Chronic gastritis
FFQ: Food Frequency Questionnaire
GCA: Gastric Cancer
IM: Internal Metaplasia
NIM: No Internal Metaplasia
WMGS: Whole metagenome shotgun sequencing
