# Supplementary figures and images for: Microbiome signatures associated with clinical stages of gastric Cancer: whole metagenome shotgun sequencing study
Source: BMC Microbiol. 2024 Apr 24;24:139. doi: 10.1186/s12866-024-03219-2 (PMC11040827; doi:10.1186/s12866-024-03219-2)

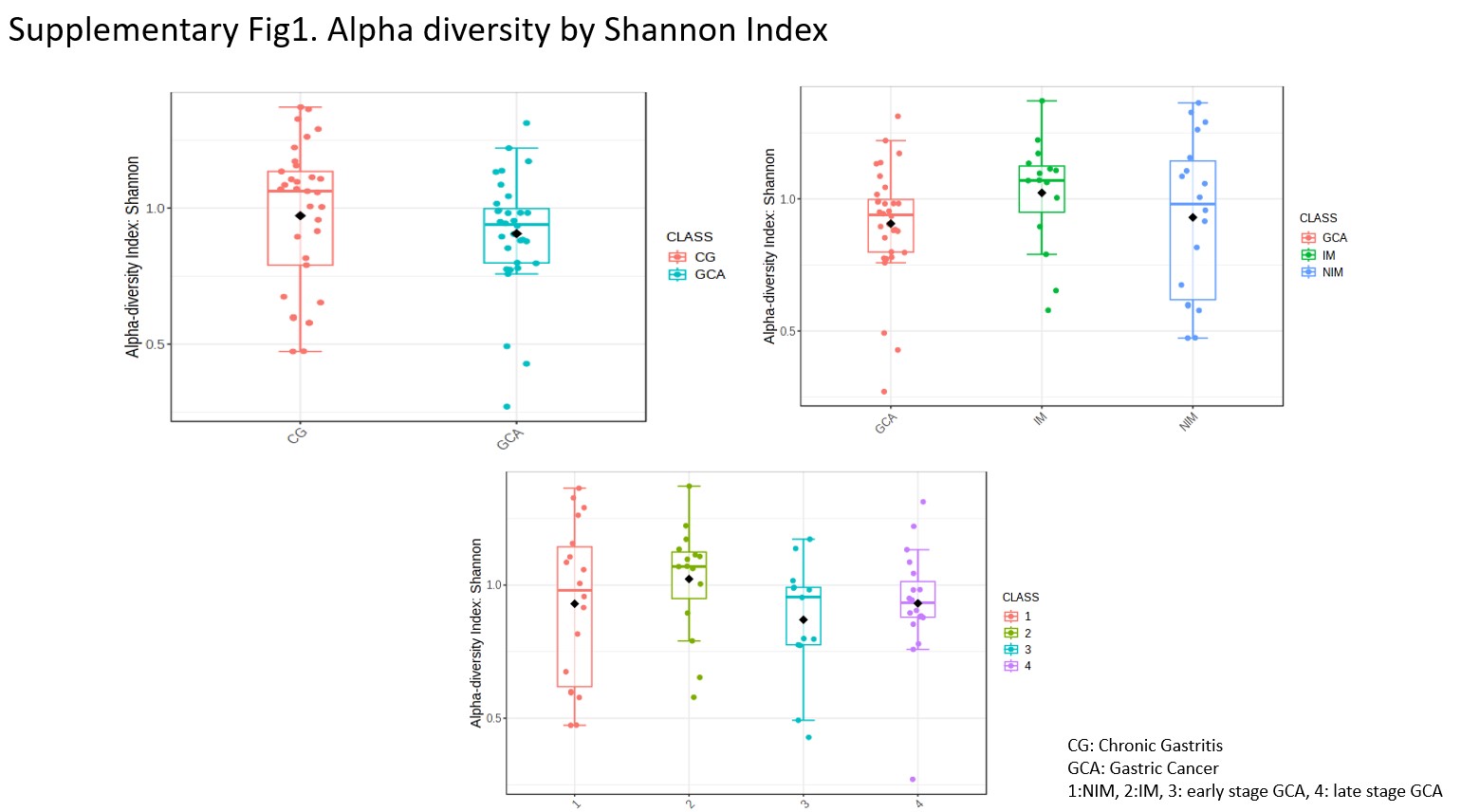

Supplement: Supplementary file 2 — Supplementary Material 2. [file 12866_2024_3219_MOESM2_ESM.jpg]

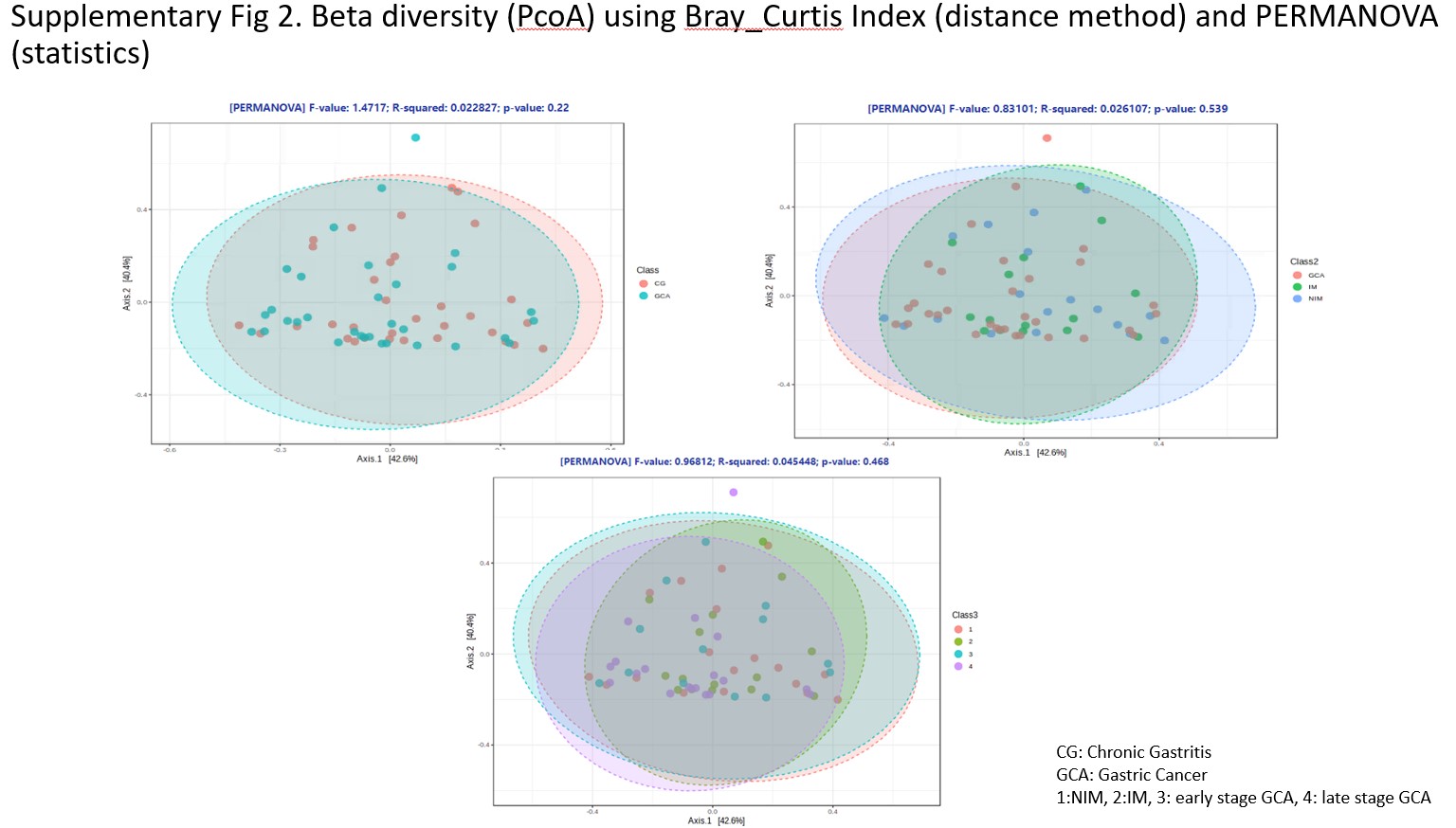

Supplement: Supplementary file 3 — Supplementary Material 3. [file 12866_2024_3219_MOESM3_ESM.jpg]

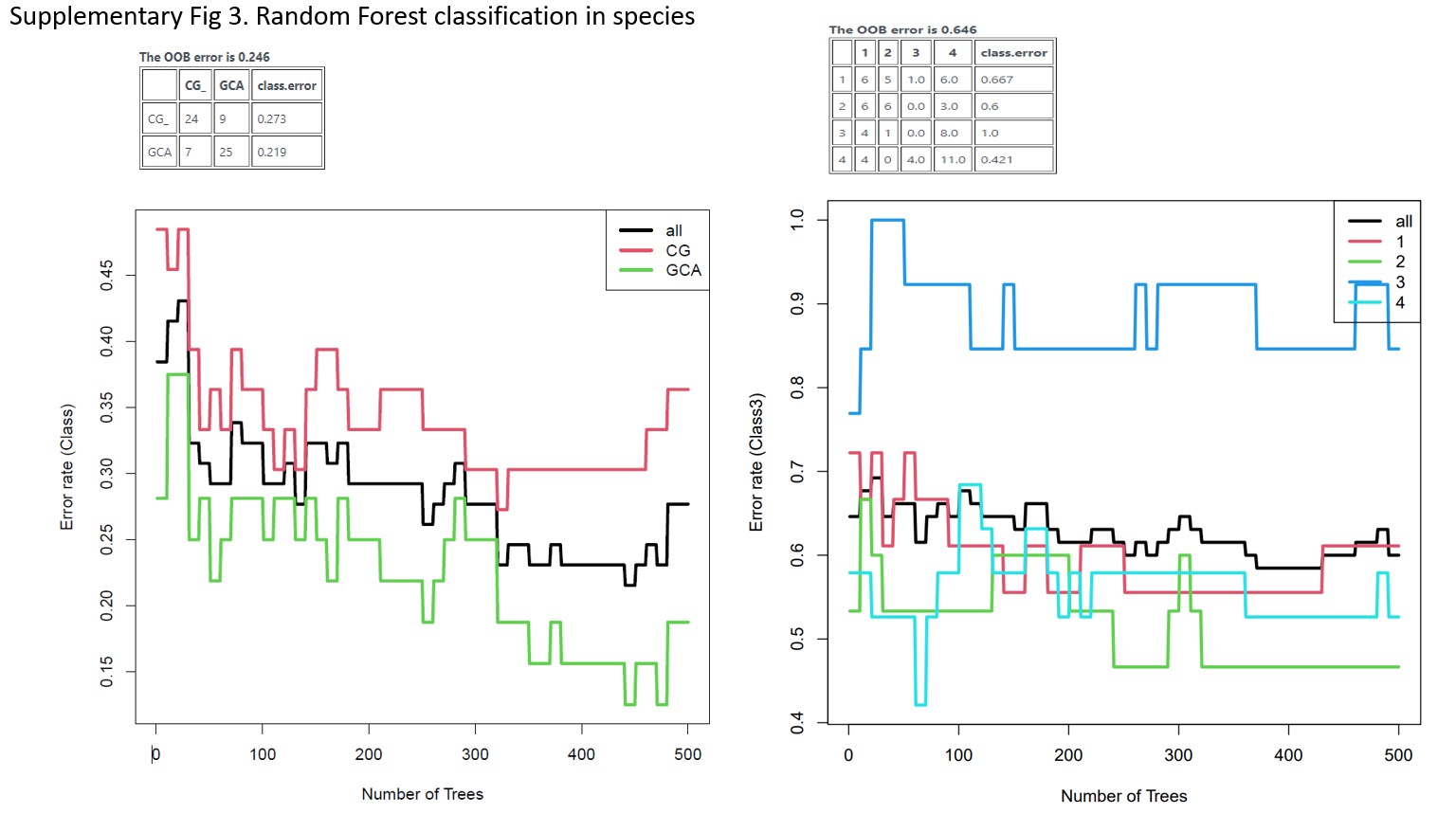

Supplement: Supplementary file 4 — Supplementary Material 4. [file 12866_2024_3219_MOESM4_ESM.jpg]
